# Correction: Partners’ experiences of their loved ones’ trauma and PTSD: An ongoing journey of loss and gain

**DOI:** 10.1371/journal.pone.0304167

**Published:** 2024-05-17

**Authors:** Rosie Powling, Dora Brown, Sahra Tekin, Jo Billings

## Notice of Republication

An incorrect version of ‘Response to Reviewers’ file was published in error. This article was republished on May 6, 2024, to correct this error. Please download this article again to view the correct version.
