# Supplementary material for: Partners’ experiences of their loved ones’ trauma and PTSD: An ongoing journey of loss and gain
Source: PLoS One. 2024 Feb 14;19(2):e0292315. doi: 10.1371/journal.pone.0292315 (PMC10866491; doi:10.1371/journal.pone.0292315)
Supplement: S1 File — This document outlines the demographic and brief contextual questions participants were asked regarding their partners’ trauma and PTSD and their relationships, and the interview schedule which was used to guide the semi-structured interviews. (DOCX) [file pone.0292315.s001.docx]

**Interview schedule**

**Demographic characteristics to be completed in person with participants:**

- Age
- Gender
- Ethnicity
- Employment
- Marital status (cohabiting/engaged/married)
- Number of children
- Ages and gender of children

**Other contextual questions:**

- What was your partner’s ‘traumatic event’ (broad description)?

*For the following questions, construct a timeline with the participant if this enables easier responding:*

- How long ago did he or she experience this event?
- When was he or she diagnosed with PTSD?
- For how long do you think he or she had actually been experiencing PTSD?
- How long had you been in a relationship with your partner when the event occurred?
- How long have you been in a relationship with your partner altogether?
- How long has your partner been seen within mental health services?

**Interview questions**

1. Would you mind telling me a bit about the circumstances in which you and your partner met?
2. How was life for you and your partner before the event happened?
3. Would you mind telling me a bit about how life is for you and your partner at the moment?
4. Looking back, how has your relationship with your partner developed since you met him/her?
5. How do you see your life in the future?
6. Overall, how do you see what has happened?
7. Would you mind telling me what treatment your partner has received and what is your opinion of the services you have both been offered?
8. If a friend of yours went through a similar experience with their partner, what advice might you give them?
9. We are coming towards the end of the interview now, how did you find the experience and how are you feeling now?
