# Supplementary material for: Partners’ experiences of their loved ones’ trauma and PTSD: An ongoing journey of loss and gain
Source: PLoS One. 2024 Feb 14;19(2):e0292315. doi: 10.1371/journal.pone.0292315 (PMC10866491; doi:10.1371/journal.pone.0292315)
Supplement: Attachment — Submitted filename: Response to Reviewers.docx [file pone.0292315.s002.docx]

Severe Mental Health Problems CBT Programme

Clinical Education Development And Research

Department of Psychology
Washington Singer Building
University of Exeter

Perry Road
Exeter EX4 4QG

Editorial Team

PLOS ONE

26^th^ May 2023

Dear Dr. Ptaszynski,

Thank you to you and your reviewers for your feedback on our manuscript **Partners’ experiences of trauma and PTSD: An ongoing journey of loss and gain (PONE-D-23-01129).**

We are pleased to provide a point-by-point response to yours and the reviewers’ comments in the table below. Please note, the page numbers referenced in the ‘Source / page number’ column refer to the appropriate page numbers when viewing the Revised Manuscript with Track Changes in the ‘Simple Markup’ format.

| Comments from Academic Editor | Response | Source / page number |
| --- | --- | --- |
| 1. Please ensure that your manuscript meets PLOS ONE's style requirements, including those for file naming. The PLOS ONE style templates can be found at [hyperlinks]. | We believe the manuscript now meets PLOS ONE’s style requirements, including those for file naming. The file name for the supplementary information has been changed to: **S1 Interview Schedule, Demographic & Contextual Questions**  We have added this caption toward the end of the manuscript, after the references:  **Supporting Information**  **S1 Interview Schedule Including Demographic and Contextual Questions**  This document outlines the demographic and brief contextual questions participants were asked regarding their partners’ trauma and PTSD and their relationships, and the interview schedule which was used to guide the semi-structured interviews. | Whole manuscript;  4 submitted files  Supplementary information  p.35-36 |
| 2. In your Data Availability statement, you have not specified where the minimal data set underlying the results described in your manuscript can be found. PLOS defines a study's minimal data set as the underlying data used to reach the conclusions drawn in the manuscript and any additional data required to replicate the reported study findings in their entirety. All PLOS journals require that the minimal data set be made fully available. For more information about our data policy, please see <http://journals.plos.org/plosone/s/data-availability>.  Upon re-submitting your revised manuscript, please upload your study’s minimal underlying data set as either Supporting Information files or to a stable, public repository and include the relevant URLs, DOIs, or accession numbers within your revised cover letter. For a list of acceptable repositories, please see http://journals.plos.org/plosone/s/data-availability#loc-recommended-repositories. Any potentially identifying patient information must be fully anonymized.  Important: If there are ethical or legal restrictions to sharing your data publicly, please explain these restrictions in detail. Please see our guidelines for more information on what we consider unacceptable restrictions to publicly sharing data: http://journals.plos.org/plosone/s/data-availability#loc-unacceptable-data-access-restrictions. Note that it is not acceptable for the authors to be the sole named individuals responsible for ensuring data access.  We will update your Data Availability statement to reflect the information you provide in your cover letter. | The original data from this study cannot be made available upon request because of the personal and sensitive nature of participants’ accounts. Audio recordings of the interviews have been destroyed in line with ethical considerations/approval from the National Research Ethics Service, relevant NHS Trust Research and Development committee, and the University of Surrey Faculty of Arts and Human Sciences. The Participant Information Sheet, which was used as part of the process of gaining participants’, and where relevant partners’, informed consent, stated that ‘Only the researcher and two Supervisors of the study will have access to the information collected.’ Therefore, if we were to share the data publicly, we would be breaching the informed consent given by participants, as well as our ethical considerations and the robust methodology upon which favourable ethical approval was based.  However, we have added this statement in the section ‘Availability of data and materials’ in order to signpost the reader if required: “To enquire about ethics and the governance review process, contact the University Ethics Committee at the University of Surrey on [ethics@surrey.ac.uk](mailto:ethics@surrey.ac.uk).” | p.36 |

**ABSTRACT**

| Comments from Reviewer #1: | Response | Source / page number |
| --- | --- | --- |
| Abstract: You state that you aim is to explore "such experiences". Please be specific. Overall, the abstract will need revision after the main manuscript has been revised. | The aim in the abstract has been clarified as suggested and parts of the abstract have been rephrased. We have also now separated the abstract into sections with subheadings. | p.2 |

**TITLE**

| Comments from Reviewer #1: | Response | Source / page number |
| --- | --- | --- |
| Title: Regarding the first part of the title "Partners' experiences of trauma and PTSD": It suggests that you are studying partners' experiences of trauma and PTSD. PTSD is a disorder, the diagnosis given to a person. However, your research is concerned with people. I suggest that you are studying the experiences partners have of being in a relationship with people who have suffered trauma and PTSD? As you even suggest in the first part of the discussion (l. 514): "This study explored how partners of individuals in mental health services experience their partners’ trauma and subsequent PTSD". Please revise accordingly. The second part of the title is intriguing. | Thank you for your feedback. We have amended the title to: ‘Partners’ experiences of their loved ones’ trauma and PTSD: An ongoing journey of loss and gain.’  We accept the point about the second part of the title as ‘intriguing’ as a compliment, thank you, and have kept this. We hope this rouses the reader’s curiosity and prompts them to read on. | p.1 title;  running title on header throughout manuscript |

**INTRODUCTION**

| Comments from Reviewer #1: | Response | Source / page number |
| --- | --- | --- |
| The review of the literature is satisfactory. However, the narrative is a bit confusing to read. Particularly the section l. 95- 102 is not coherent, confusing to read, and does not contribute to stating the knowledge gap. Please revise to clarify. | Thank you for this feedback. We have deleted that paragraph and revised and relocated this information in previous paragraphs in order to be more coherent and to emphasise more clearly the knowledge gaps in the current literature. | p.3-5 |
| In l. 102-105 is not supported by evidence; a reference is lacking. | We have added a reference (reference 21). | p.5 |
| The research question and aim are unclear. In revising according to the following comments, it may be useful for you to apply the PICo mnemonic in structuring your research question and aim: • You state that partners' experiences of trauma and PTSD is the focus of the research. In the same way as in the title this wording suggests that you are studying partners' experiences of symptoms and diagnosis, rather than studying experiences of being in a relationship with a person with PTSD. Reading your introduction, it seems that you are interested in partners' experiences of living together with a person with trauma and PTSD; or partners' experiences of the relationship? | We have used the PICo mnemonic to reframe the research question to: ‘What are the experiences and views of people who have a partner with a history of trauma and diagnosis of PTSD?’  P - people who have a partner  I – experiences and views  Co – the partner has a history of trauma and a diagnosis of PTSD  The ‘experiences’ we were interested in was purposefully broad so as not to constrain participants’ answers to the interview questions which asked about a range of experiences. Therefore, in line with the research aims and guidelines for qualitative research, we have made the decision to maintain ‘experiences’ in broad terms, without putting further parameters on it. | p.5 |
| Further, introduction is missing to the key subjects; trauma and PTSD. Are they the same? Or how does trauma develop into PTSD? To be part of your study should the person who experiences a trauma be diagnosed with PTSD? | Thank you for pointing out this ambiguity. We have amended the inclusion criteria to make it clearer that to be part of the study, the person who experienced the trauma did need a diagnosis of PTSD. | p.6 |
| You state that there is a significant gap in the literature in exploring the perspectives of partners from outside of high-risk occupational settings. However, your research questions do not reflect this gap. Further, your focus on this particular group should be reflected in the "Participants" section. | That is correct, the research question does not specifically seek to redress this gap in the literature, as we did not *exclude* partners of people in high-risk occupations. However, we sought to include a wide range of potential participants whose partners had experienced different types of traumas.  We have added this sentence to the Participants section: ‘The study was interested in individuals’ experiences irrespective of their partner’s occupation, and sought to include a range of participants whose partners had experienced different types of trauma.’ | p.6 |
| • In l. 141 you introduce the term "service user" for the first time. Who are they? In what context are they service users? This term should be clarified in the introduction.  I wonder whether rewriting "service users" would account for the Context part of the PICo mnemonic in describing the research question? | In this context we have used the term ‘service user’ to describe people who have a history of trauma and are awaiting or receiving psychological treatment for PTSD in a specialist Trauma Service. They are service users in that they are receiving services from the NHS trauma service through which we recruited. That they were service users was not a primary concern of the study, but we required people to have received a diagnosis of PTSD for their partners to be eligible, which was achieved by recruiting through a service where clinical diagnoses had been made. We have amended the Participants section to clarify this and clarify the term ‘service user’ and how it has been used in this context.  Thank you for the suggestion about using the term ‘service user’ in the Introduction and research question. We have decided not to adopt this approach, because it was not the fact that someone’s partner was a service user that made us interested in their experiences. It was that their partner had a history of trauma during the relationship, and was experiencing PTSD. Because we recruited through an NHS service, participants’ partners were service users but this was not a research aim as such. Also, research indicates that a lot of people who use services do not favour the term ‘service user’ so we have endeavoured to minimise its use. | p.6 |
| • Concerning the language: l. 107 you write: "… with little qualitative research hearing partners’ experiences and views in depth." Setting out for the formulation of the aim, "hearing" seems a bit odd. In qualitative research the aim is to explore, understand, investigate. In describing the aim, you use "participants" (l. 112). Please, reformulate so the aim reflects the population. Overall; the use of parenthesis is confusing: do they signal that their contents are less important? In which case it could be left out of the text. Particularly in two places the problems / symptoms of the person with PTSD is put in parenthesis. These experiences are very important to be able understand the situation of the partners. A short introduction to the person who has been diagnosed with PTSD, is needed to fully understand the challenges meeting the partners. | Lines 107 and 112 (original line numbers) have been revised.  We have revised those sentences to point out their importance. | p.4 and p.5 |
| In l. 48-49 you write that perceived social support is protective factor in the development of posttraumatic stress disorder. This is a bit confusing; it indicates that social support is particularly important after having had a trauma to avoid developing PTSD. While it seems that your study is focusing on being in a relationship with a person who has developed PTSD? | According to Ozer at all., (2003), social support is not only protective against developing PTSD, but also, individuals who experience more social support after a traumatic event tend to experience lower levels of PTSD symptoms or rates of current PTSD. We have clarified this. | p.3 |
| l. 50-51: "…the impact on those providing such support…" –The impact of what? What is it that impacts on the person providing support? Please clarify. | Thank you for your comment. We have expanded the information to address your comments. | p.3 |
| l. 52-53: "…a limited number of studies have explored how trauma affects partners and relationships". What trauma are you writing about? The partners of whom? Do you mean being in a relationship with a person who has experienced a trauma – or who has developed PTSD? Please, clarify. | It has been revised as “…relatively few studies have explored the impact of a person’s trauma and PTSD on their partner and relationships”. | p.3 |
| L. 53-58: "Research has tended to use quantitative methodology…": the research you are referring to actually used quantitative methodology. Writing about tendencies is a bit odd, when you want to introduce the knowledge we have gained from quantitative research. | Thank you for your feedback. We have revised this section according to your feedback. | p.3 |
| l. 79: "This research reports similar deleterious effects of being the partner of …". Similar to what? | This has been revised as “This body of research also indicates deleterious effects of being the partner of an individual with PTSD …” | p.4 |
| L. 80: "Someone": I suggest you write "a person". | Thank you for your suggestion. We have amended this. | p.5 |
| In terms of content, the introduction is long with the use of redundant words. I suggest a revision focusing up on tightening up the language; i.e. less referring and more directly stating the results. Three examples with suggestions: • L 52: "To date, a limited number of studies have explored how trauma affects partners and relationships. Research has tended to use quantitative methodology and focus on 54 specific traumas and populations at high risk of exposure to trauma (most commonly 55 combat-related trauma in male military personnel and veterans)". A more focused version e.g. could be: "Few studies have explored how trauma affects partners and relationships. Quantitative research has focused on specific traumas and populations at high risk of exposure to trauma, mainly combat-related trauma in male military personnel and veterans. • l. 62: Male soldiers’ symptoms (particularly sleep problems, dissociation and sexual problems) have been demonstrated to predict lower relationship satisfaction for both soldiers and their female partners". A more focused version e.g. could be: "For male soldiers’ and their partners' symptoms such as sleep problems, dissociation and sexual problems can predict lower relationship satisfaction." • l 77: "An even smaller body of literature has qualitatively explored partners’ experiences, with what little research that has been conducted again mostly being with spouses of workers in high-risk occupational groups". A more focused version e.g. could be: "Few qualitatively studies explored partners’ experiences. Most studies concerned spouses of workers in high-risk occupational groups". In the section l. 79- 85 you repeatedly describe qualitative studies reporting on effects and outcomes. I suggest revising the text so that it reflects the reference to qualitative research. | Thank you for these helpful suggestions. We have revised the introduction, including these sections, to be more concise and consistent. | p.3-5 |
| Examples: • l. 80: "… similar deleterious effect of being a partner...". I suggest you describe how being a partner was deleterious (l 80). | Thank you for this suggestion. We have elaborated on this point. | p.4 |
| • l. 81: "In one qualitative study exploring heterogeneous traumas, a history of trauma in one or both partners had both positive and detrimental effects on interactional patterns in the relationship, including increased and decreased cohesion, communication and understanding". To highlight experiences / perceptions this you "A history of trauma in a study exploring heterogeneous traumas was reported to both increase and decrease experiences of cohesion, communication, and understanding in partners' interaction" | Thank you for your suggestion. We have amended this point. | p.4 |

**METHOD**

| Comments from Reviewer #1: | Response | Source / page number |
| --- | --- | --- |
| Participants l. 127: use the abbreviation PTSD | We have decided to keep this written out because it is the start of a sentence. Whilst we cannot see any advice in the PLOS ONE formatting guidelines about whether or not to abbreviate at the start of the sentence, we understand it is usually considered preferable to start a sentence with an acronym or abbreviation written out in full. | p.6 |
| l. 127-128: What is a "type 1 trauma"? A definition is lacking. | We have added ‘single-incident’ into the definition and removed the parentheses to make the definition clearer, plus added a reference. | p.6 |
| Procedure l. 135: "A Service User and Carer panel at the University of Surrey 136 was consulted regarding ethical considerations". These considerations are important. Please, elaborate. For example, I expect that the lengthy individual interviews have called for certain considerations? | Thank you for your suggestion. We have elaborated upon this.  The length of the interviews was not raised by the panel (being an in-depth IPA study this was anticipated), but they made certain suggestions. For example, in line with their feedback we included a question in the interview schedule asking how the interview had been for participants, managing any distress, and signposting if necessary (including to local organisations and the GP) in order to support the wellbeing of participants. | p.6-7 |
| l. 141: How and by whom were potential participants who had not been in direct contact with the Trauma Service approached (after informed consent from service users were obtained)? | These participants were invited to participate by their partner. We have clarified this. | p.7 |
| l. 146: To make visible the researchers (first authors) pre-understandings, please elaborate on relevant characteristic; i.e. qualifications, experiences. | We have added information regarding the first author’s qualification, experiences and interests. | p.7 |
| l. 149: You use terms such as "pre-determined" and "open questions". Are these terms referring to IPA? If not, I suggest you use well-established concepts such as "semi-structured interview guide" and "open-ended. | Thank you for your suggestions. We have amended this, whilst also retaining the previous adjectives in line with IPA. | p.7 |
| l 153-156: rather than referring the guidelines suggested by Yardley, briefly describe measures to comply with relevant parts of these guidelines | We have expanded this section, adding examples of the measures taken in line with each of the 4 principles suggested by Yardley. | p.8 |

**RESULTS**

| Comments from Reviewer #1: | Response | Source / page number |
| --- | --- | --- |
| To provide readers with an overview, I suggest to display characteristics of the participants in a table and briefly present characteristics in text. | Thank you for your suggestion; we have included a table of participants’ characteristics and condensed the brief description in the text. | p.9-11 including Table 1. Participants’ Sociodemographic Information |

**DISCUSSION**

| Comments from Reviewer #1: | Response | Source / page number |
| --- | --- | --- |
| In places you emphasize that experiences related to i.e. "some participants" or "a couple of participants". This should be rephrased to discuss the topics in more general terms. | These sections have been rephrased to discuss the topics in more general terms.  We have also rephrased some similar phrases in the Results section to enhance readability of the results. | p.24-25  p.12-24 |
| l. 524-527: To better understand the needs of the partners it would be helpful if you briefly elaborated on the concept "assumptive world". | This paragraph has been expanded and restructured to elaborate on this. | p.24-25 |
| l. 544 – 547: I suggest to discuss and highlight part that are particular interesting in relation to professional practice. | Our apologies but we are unsure what the reviewer means by this comment in relation to this section: *“Consistent with previous research in partners of people with PTSD [34], participants reported experiencing a vast array of emotions. These emotional and psychological consequences of the trauma were akin to the impact described in the literature on* *carer burden in psychosis [35,36] and dementia [37].”*  Nevertheless, we have tried to highlight how our results impact professional practice through the ‘Implications for Services’ section. | p.26-27 |
| Implications for services l. 585: The chain of argument to conclude that "…the issue of considering partners is not simply a systemic issue, but a moral, political and financial one" is insufficient. Regarding the financial aspect, I suggest to provide some background in the introduction. | We have removed this sentence. | p.27 |
| Strengths and limitations The researcher's pre-understanding is of great importance for the credibility of data and should be addressed. | The first author’s pre-understanding is outlined to help the reader judge the credibility of the data, including how the research was shaped by the researcher’s background, assumptions, prior knowledge and clinical experiences. | p.28 |
| Conclusions l. 613-615: This part of the conclusion is not supported by the results. | We have made this point with less emphasis on the experiences of partners of people with high-risk occupations. We believe this point about giving voice to partners, who are often overlooked in research and clinical services, and shedding new light on the impact of being a partner of someone who has experienced a trauma and is diagnosed with PTSD is important and in line with the study’s findings. | p.29 |

Comments from Reviewer #2:

| Comments from Reviewer #2: | Response | Source / page number |
| --- | --- | --- |
| all participants of the study identified as white so how do you then call for The ‘development of culturally-sensitive intervention’? This does not seem to have a very direct connection here. Can your implications further target religious or faith-based approaches also? | Thank you for raising this discrepancy. We have clarified this point to make it clear that further research is required with a more diverse sample to inform culturally-sensitive services.  We have also removed the word ‘culturally-sensitive’ in ‘Implications for services’, given the findings of this study are not able to inform the cultural-sensitivity of any interventions. | p.29  p.26 |
| since participants talked about the desire to have a group to discuss their partners’ PTSD, how about service providers introducing (support the creation of)safe spaces or networks to discuss these among partners? | Thank you for your suggestion which we have included. | p.27 |

Thank you again for considering our manuscript and providing such comprehensive feedback. We hope these amendments address the points raised and we look forward to hearing further from you.

Yours sincerely,

Dr. Rosie Powling

Clinical Psychologist & BABCP Accredited Cognitive Behavioural Therapist

Clinical Education Development And Research, University of Exeter
