# Supplementary material for: Partners’ experiences of their loved ones’ trauma and PTSD: An ongoing journey of loss and gain
Source: PLoS One. 2024 Feb 14;19(2):e0292315. doi: 10.1371/journal.pone.0292315 (PMC10866491; doi:10.1371/journal.pone.0292315)
Supplement: Attachment — Submitted filename: Response to Reviewers.docx [file pone.0292315.s003.docx]

Severe Mental Health Problems CBT Programme

Clinical Education Development And Research

Department of Psychology
Washington Singer Building
University of Exeter

Perry Road
Exeter EX4 4QG

Michal Ptaszynski

Academic Editor

Editorial Team

PLOS ONE

30^th^ August 2023

Dear Dr. Ptaszynski,

Thank you to you and your reviewers for your feedback on our amended manuscript **Partners’ experiences of their loved ones’ trauma and PTSD: An ongoing journey of loss and gain (PONE-D-23-01129R1).**

We are pleased to provide a point-by-point response to the comments in the table below. Please note, the page numbers referenced in the ‘Source / page number’ column refer to the appropriate page numbers when viewing the Revised Manuscript with Track Changes in the *‘All Markup’* format.

| Comments | Response | Source / page number |
| --- | --- | --- |
| Please review your reference list to ensure that it is complete and correct. If you have cited papers that have been retracted, please include the rationale for doing so in the manuscript text, or remove these references and replace them with relevant current references. Any changes to the reference list should be mentioned in the rebuttal letter that accompanies your revised manuscript. If you need to cite a retracted article, indicate the article’s retracted status in the References list and also include a citation and full reference for the retraction notice. | The reference list has been checked to ensure it is complete and correct. No references have been removed. Any references that have been reordered are in line with the reviewer’s feedback to amend sections of text. Specific changes to the reference list are made clear in this rebuttal letter. | p.31-37 |
| Reviewer #1: The authors have done a great job in improving the manuscript. However, some work still needs to be done.  Abstract: The abstract has become clear and easy to read. One thing should be amended: l.32 is part of the methods. Please, move to the Methods section of the abstract. | Thank you for this feedback.  This line has been moved to the Methods section of the Abstract. | p.2 |
| Introduction: The section has improved; however, the section is lengthy and the narrative still is somehow confusing to read. While the study is qualitative focusing on how better to understand people´s experience, the language in places should be rephrased to give a sense of coherence throughout the manuscript. Particularly on page 3 l. 61- 71, the use of nomenclature from quantitative research is disturbing, i.e. "correlation", and "effect size". I suggest a revision. In particular, in l. 69-71 the point is hard to see. I suggest you revise to highlight the focus on people with PTSD and partners rather than effect size. An example of revising l. 61- 64 could read: "Further in veterans with more severe PTSD symptoms, their partners have increased distress, lower levels of functioning and poorer mental health". | Thank you for your feedback. We have amended this section and removed the nomenclature from quantitative research. | Introduction, especially p.3-4 |
| In places, too much details makes the reading un-interesting without adding relevant knowledge. This pertains to highlighting different sub-groups of high-risk occupational groups; or the highlighting of the amount of research. One example is l. 72-75, that could be shortened to "Partners of fire-fighters experience similar symptoms such as major depression, panic disorder, and generalised anxiety disorder [10] and more recently, 72.5% of spouses of 76 doctors reported psychological distress during the COVID-19 pandemic [11]". Another example is l. 99-100: "…recruited 100 from a university-based counselling centre…": I do not see the relevance of this information; and I suggest you revise l. 99-103 to make your point clear. | Thank you for your feedback. We have amended the Introduction section in line with this feedback, including your specific suggestions. | Introduction, especially p.4-5 |
| Further, unnecessarily referring to existing research disturbs the reading; in many instances the reference is sufficient to show the evidence. Here are some examples: • l. 52: "Meta-analyses have established that" - can be removed. • lines 60 & 64 & 68 & 86: "have/has been shown to". I suggest you remove and where necessary rephrase. • l 62: "Research has established": Remove and rephrase accordingly • l. 94: "in one such qualitative study" – can be deleted. | L52 – This phrase has been removed.  L60 – This has been rephrased to ‘can’.  L64 – This sentence has been deleted and the reference incorporated into a previous citation (reference 5), because it didn’t add any additional information. The references and reference list have been reordered accordingly.  L68 - This has been rephrased to ‘have been linked.’  L86 – This has been rephrased to ‘might’.  L62 – This has been removed and rephrased in line with the feedback.  L94 -This has been rephrased to make the sentence clearer and ensure the sentences within the paragraph flow. | Introduction p.3-5; references p.32 |
| Finally, unnecessary small words are used throughout the manuscript, making it longer than necessary and in places more confusing to read, for example: • l. 55: "such": you indicate, that one specific earlier defined kind of social support is referred to. suggest you delete. • L.56: "relatively": in relation to what? suggest you remove • "Also": is used comprehensively throughout the manuscript, in places it has no meaning (i.e. l. 37, l. 82 and l. 458) and in places it is unclear what it is referring to (i.e. l. 79) Please go through critically. • l. 565 "aforementioned" – suggest you delete | Thank you for your feedback. We have read through the manuscript critically and removed unnecessary words, and rephrased some sections to shorten them.  L55 – ‘Such’ has been deleted.  L56 – ‘Relatively’ has been deleted.  We have removed the word ‘also’ where redundant, including the suggested lines 37, 82, 458 and 79.  L565 – ‘Aforementioned’ has been deleted.  In addition, the words ‘meaning-making’ (p.6) and ‘same-sex’ (p.28) have been hyphenated to ensure they are consistent with the spelling where else they appear in the text. | Whole manuscript  p.6  p.28 |
| Other comments: • L. 93: I do not understand that the experiences are "indicated"? The experiences referred to are, I guess, results from the studies? • l. 106-108: It seems that some argument is missing in this sentence; Development of knowledge and understanding cannot in itself "increase partner's social connectedness …". | L93 – The experiences referred to are indeed the results of the studies. We have changed the word ‘indicated’ to ‘described’ to make this clearer.  L106-108 – This has been rephrased with the argument made clearer. According to this study's findings, due to the lack of understanding about partners' experiences, partners can feel alone and invisible. | p.5  p.5-6 |
| Methods The section is in some places still referential, without contributing insight into the actual study, particularly in the section on credibility. l 152: "was necessary" and l. 153: was required. Please, reformulate to describe what you actually did (i.e. consent was obtained) | We have reformulated these sentences in line with your feedback, thank you. We have also clarified how participants were provided with an information sheet in this section. | p.8 |
| l. 163-166: "A semi-structured interview schedule …. explored experiences of". An interview guide cannot explore; it is a means used to explore something. Please amend | We have amended this in line with your feedback, thank you. | p.8 |
| L. 166: The sentence is repetitive, please delete | This sentence has been deleted. The references and reference list have been reordered accordingly. | p.8, p.34 |
| l. 173-175: can be deleted | This sentence has been deleted. | p.9 |
| l. 177: "It is argued that": Please delete. You actually supported commitment and rigor. | This phrase has been deleted. | p.9 |
| l 187-189: can be deleted | This sentence has been deleted. | p.9 |
| l. 189-191: If you want to keep this, it should be part of the discussion or conclusion. | Thank you for your feedback; this sentence has been moved to the Conclusions section.  In addition, the full spelling of the acronym ‘NHS’ has been changed to spell it out the first time it appears in the text, as opposed to the second time. | p.30  p.6-7 |
| Strength and limitation: l 636-650: I do find this section to be too long and unnecessarily detailed and in places even referential (i.e. l. 637-640). With the detailed description in the methods section it is sufficient to write more concisely, for example l 640-642 could be abbreviated to "The first author's preunderstanding…". Please, revise. | Thank you for your feedback. The ‘Strengths and limitations’ section has been amended and condensed, including some reordering to improve its flow.  L640-642 has been amended in line with your feedback, thank you.  The analysis section of the Method has been amended to clarify that other members of the research team read portions of the transcripts and coding. This was to ensure that the relevant comment in the ‘Strengths and limitations’ section is not new information regarding the method. | p.28-30  p.28-29  p.10 |
| Conclusion: This sentence does not make sense: "… one overarching theme emerged that encapsulated the data, that is participants experienced trauma and PTSD as an ongoing journey of loss and gain". Please amend. | This sentence has been amended; thank you for your feedback. | p.30 |

Thank you again for considering our manuscript and providing such comprehensive feedback. We hope these amendments address the points raised and we look forward to hearing further from you.

Yours sincerely,

Dr. Rosie Powling

Clinical Psychologist & BABCP Accredited Cognitive Behavioural Therapist

Clinical Education Development And Research, University of Exeter
